# The Visible-Light-Driven Activity of Biochar-Doped TiO_2_ Photocatalysts in β-Blockers Removal from Water

**DOI:** 10.3390/ma16031094

**Published:** 2023-01-27

**Authors:** Agata Kowalczyk, Bożena Zgardzińska, Karol Osipiuk, Katarzyna Jędruchniewicz, Katarzyna Tyszczuk-Rotko, Magdalena Goździuk, Haitao Wang, Bożena Czech

**Affiliations:** 1 Institute of Physics, Maria Curie-Sklodowska University, 20-031 Lublin, Poland; 2 Department of Radiochemistry and Environmental Chemistry, Institute of Chemical Sciences, Faculty of Chemistry, Maria Curie-Sklodowska University, 20-031 Lublin, Poland; 3 Department of Analytical Chemistry, Institute of Chemical Sciences, Faculty of Chemistry, Maria Curie-Sklodowska University, 20-031 Lublin, Poland; 4 MOE Key Laboratory of Pollution Processes and Environmental Criteria, Tianjin Key Laboratory of Environmental Technology for Complex Trans-Media Pollution, Tianjin Key Laboratory of Environmental Remediation and Pollution Control, College of Environmental Science and Engineering, Nankai University, Tianjin 300350, China

**Keywords:** photocatalysis, drugs, metoprolol, propranolol

## Abstract

Water is the most important life-giving resource on earth. Nowadays, intensive growth of the world population has resulted in increased water consumption and the production of wastewater. Additionally, the presence of pharmaceuticals in treated conventional wastewater or even in the environment is strictly indicating that present techniques of wastewater treatment are not efficient enough and are not designed to remove such pollutants. Scarce water resources in the world are the main driving force for the innovation of novel techniques of water and wastewater treatment. Photocatalysis, as one of the advanced oxidation processes, enables the transformation of recalcitrant and toxic pollutants into CO_2_, water, and inorganic salts. In the present paper, the photocatalytic oxidation of β-blockers—metoprolol and propranolol—are described. For photocatalytic oxidation, novel TiO_2_ photocatalysts modified with biochar were used. Photocatalysts were prepared by sol-gel method and the effect of photocatalysts type, presence of inorganic ions, dissolved organic matter, and different water matrix was established. The results indicate that using only the decrease in the tested pollutant concentration is not effective enough in establishing the treatment method’s safety. There is a need to use additional testing such as ecotoxicity tests; however, the key parameter is the properly chosen tested organism.

## 1. Introduction

**Figure 1:**
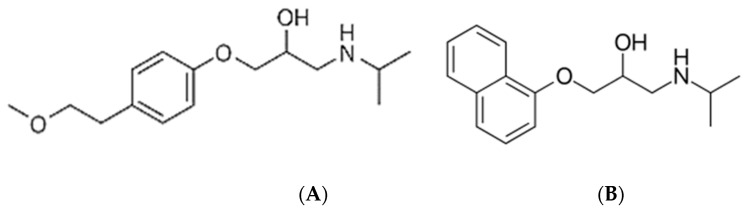
β-blockers (**A**) metoprolol, (**B**) propranolol.

Water is the most important substance on the earth [1]. Nowadays, intensive growth of the world population has resulted in increased water consumption and production of wastewater [2]. Additionally, the extension of life results in the consumption of more and more drugs [3]. Pharmaceuticals and personal care products (PPCPs) are gaining interest recently as emerging pollutants [4,5]. Their identification in wastewater [4], freshwater [6], or even groundwater [7] is evidence that nowadays existing wastewater treatment methods are not effective in the removal of such compounds. These low removal rates may arise from the fact that PPCPs are designed to reveal bioactivity and may interact with organisms in activated sludge [8]; metabolites may reveal higher bioactivity than the parent compound [9], or some conjunctions of metabolites towards parent PPCPs are observed [10]. Although their presence in the environment is evidenced and monitored, their effect on living organisms is not fully understood [11]. One of the most noted PPCPs besides nonsteroidal anti-inflammatory drugs, antibiotics, and hormones are β-blockers. Among β-blockers, metoprolol (Met), propranolol (Pro) (Figure 1), atenolol, esmolol, sotalol, nadolol, and timolol are classified and used in cardiology (regulation of heart rhythm and blood pressure) [12].

Due to the hydrophilicity of atenolol, nadolol, and sotalol, as well as their low metabolism rate and high half-life, they are present in the wastewater in the parent form whereas lipophilic Pro, Met, or timolol are present in the wastewater in the form of metabolites. The concentration of β-blockers in the surface water may reach several ng/L or µg/L [13]. In the wastewater treatment plant, metoprolol undergoes O-dealkylation, and metoprolol acid is a major intermediate, whereas propranolol biotransformation was lower, and 4-hydroxyphenyl acetic acid is noted as a by-product [13].

Advanced oxidation processes (AOPs) that are based on the generation of highly reactive species (*OH, 1.8–2.7 V, t_1/2_ = 20 ns) [14] are known as very effective in the removal of many recalcitrant pollutants from water, including PPCPs [15]. Among AOPs, photocatalysis utilization of visible light has gained great attention recently [16]. The traditionally used TiO_2_, besides high activity, stability, and chemical indifference, requires highly energetic UV light [17]. The approach to increase the activity of TiO_2_ towards visible light considers doping with metals [18], non-metals [19], formation of heterojunctions [20], or sensitization with carbonaceous materials [21]. Doping with C promotes the charge transfer from the bulk TiO_2_ to its surface inducing visible-light-driven photocatalytic activity but also increasing the available surface for adsorption (initial step of photocatalytic reaction) and oxidation [22]. The main advantage of carbonaceous materials is the possibility to apply environmentally friendly or waste-derived materials. The studies of Wang et al. [23] describe the removal of enrofloxacin over biochar-modified TiO_2_; however, the authors concentrated on the UV activity of the photocatalysts (UV lamp 254 nm was applied in the tests). Biochar is a carbonaceous material obtained after the pyrolysis of biomass in an oxygen-free atmosphere [24].

The unquestionable advantages of AOPs are their high efficiency in a short time, and the decomposition of highly persistent compounds; however, during oxidation, some by-products can be created revealing high toxicity. Thus, the effectiveness of AOPs could be established not only in the form of target pollutant removal but also the toxicity of AOP-treated samples should be determined [25]. The properties of biochar e.g., surface area, pH, quantity, and quality of surface functional groups are governed by applied feedstock and pyrolysis temperature [26]. Thus, in the presented manuscript three various biochars were applied for doping of TiO_2_. The presented studies aimed to establish the following: (i) the visible light photocatalytic activity of the obtained photocatalysts in the removal of two common β-blockers: metoprolol and propranolol from water; (ii) the effect of matrix parameters: the presence of inorganic anions, dissolved organic matter, or different types of water (distilled, tap water, or treated wastewater); and (iii) estimation of the toxicity of water before and after photocatalytic oxidation using plants and bacteria.

## 2. Materials and Methods

### 2.1. Catalysts Preparation

The reagents were of analytical grade and used without further purification. Titanium(IV) butanoate, tetraethoxysilane, ethanol, butanol, sodium chloride, sodium nitrate, and sodium carbonate and supplied by POCH (Poland), whereas metoprolol, propranolol, and tannic acid were purchased from Merck (Poland).

The photocatalysts were obtained by sol-gel method using titanium(IV) butanoate (TBOT) as the source of Ti. The control of the process of TiO_2_ crystallization (small and uniform crystals) was performed using tetraethoxysilane (TEOS) according to the procedure described in [27]. Briefly, after sonication (10 min) the mixture of TBOT (30 mM) and TEOS (7.5 mM), biochar was added. Hydrolysis was performed using a mixture of ethanol and butanol. After the hydrothermal treatment at 60 °C for 24 h, the formed solid was washed with distilled water and dried for 12 h at 110 °C. The activity of TiO_2_ towards visible light was enhanced using a 10% addition of 3 types of biochar during photocatalysts preparation: biochar from hardwood, from sunflower, and softwood (pyrolyzed at 600 °C in the N_2_ atmosphere), thus the photocatalysts were labeled as follows: TB1, TB2, and TB3, respectively.

### 2.2. Catalysts Characterization

For the determination of the surface area and porosity, the low-temperature N_2_ adsorption (ASAP 2420 Micromeritics, Norcross, Georgia, USA surface area, and porosity analyzer) was applied. The morphology of photocatalysts was determined by scanning electron microscopy (SEM) VEGA3 TESCAN equipped with an EDS detector (Tescan, Brno, Czech Republic), in high vacuum mode at the accelerated voltage of 10 kV or 20 kV. Spectroscopic studies: XPS (UHV Prevac sp. z o.o., Rogów, Poland), and FT-IR (Nicolet 8700A Waltham, MA, USA) were used for photocatalysts’ surface characterization.

For the analysis of the structure and properties: defects, size of free volumes, particle packing efficiency positron annihilation lifetime spectroscopy (PALS). The PALS study used the interaction of positron (e+) and positronium (Ps) probes with the molecules of the medium, taking into account various photocatalysts and oxidation stages (darkness and lighting conditions of the samples).

The 0.8 MBq ^22^Na positron source in a Kapton^®^ envelope and the liquid sample were placed in a measuring chamber. The sample was measured under conditions of temperature control and stabilization and sample illumination (through a beryllium window). The spectra were registered using a digital fast-slow coincidence spectrometer with the time resolution FWHM = 190 ps. For each of the catalysts, one spectrum was collected in the dark, and then on the same sample—a one-hour spectrum for the illuminated sample. The PAL spectra were analyzed with LT 9.2 software [28], distinguishing components correlated with the annihilation of free positrons (τ_2_ ≈ 0.42 ns) and the positronium atom in the singlet (p-Ps) and triplet (o-Ps) states. The last component is sensitive to the size of the free volumes present in the material and the o-Ps lifetime is used to determine the radius [29,30] of the free volume, e.g., defects, spaces around the molecules, pores, etc. In the tested systems, free volumes are bubbles in the liquid fraction of the sample [31,32,33]. The radius of the bubble expressed in the o-Ps lifetime depends, among others, from surface tension, external pressure, dielectric constant, viscosity, and impurities (presence of trace compounds) in the liquid.

### 2.3. Metoprolol and Propranolol Removal from Water

The photocatalytic activity of the photocatalysts (0.5 g/L) was estimated in the removal of Met and Pro (10 mg/L) from water. The process was conducted in the photochemical reactor (0.7 L) with a Vis lamp placed vertically in the center. The photocatalytic process was preceded by 30 min of dark sorption to maintain the sorption–desorption equilibrium. Then the samples were collected after 5, 10, 15, 30, 45, and 60 min of irradiation. The amount of Met and Pro decomposed over photocatalysts was estimated on the concentration loss, expressed as c/c_0_ ratio, where c is the actual concentration and c_0_ is the initial concentration. The concentration of Met and Pro was estimated using UV-Vis spectrophotometry (Specord 200, Analytik Jena; scan rate 600 nm/min; time response: 0.1 s; spectral band:2 nm) and λ_Met_ = 222 nm and λ_Pro_ = 290 nm, respectively, using calibration curves (R^2^_Met_ = 0.9989, R^2^_Pro_ = 0.9978). The effect of various parameters: the presence of inorganic ions (using 0.001 M NaCl, NaNO_3_, Na_2_CO_3_) and dissolved organic matter (DOM) (with tannic acid as the representative at a concentration of 0–100 mg/L) and the effect of water matrix (e.g., distilled water, conventionally treated wastewater, river water from Dniepr river in Nahirne, Ukraine) was also established using TB2 photocatalysts and 60 min of the oxidation. The river water sample was taken following the water and sewage company guidelines for collecting water samples for physicochemical tests. Water for testing was taken on 23 October 2021, into a sterile plastic bottle (no additional filters were used), 1 m from the shore from a depth of 30 cm. The sample was taken from the mainstream of the Dnieper River, near Nahirne in the Kirovograd Oblast in Ukraine (49°05′22.5″ N 33°02′51.4″ E). The bottle was transported in the dark at 4–6 °C. From the time of sampling from the river until the measurements, the water was stored in a refrigerator at +4 °C.

The toxicity of water before and after the photocatalytic treatment was established using PHYTOTOXKIT with *Lepidium sativum* L. as the tested organism. In the test, the germination process and early plant development with the average root length, are determined [34]. Simultaneously, to verify the effect of photocatalytic treatment on the water organism, the Microtox^®^ test was performed using standard operation procedure and *Allivibrio fischeri* as the tested organism [35].

## 3. Results

**Figure 2:**
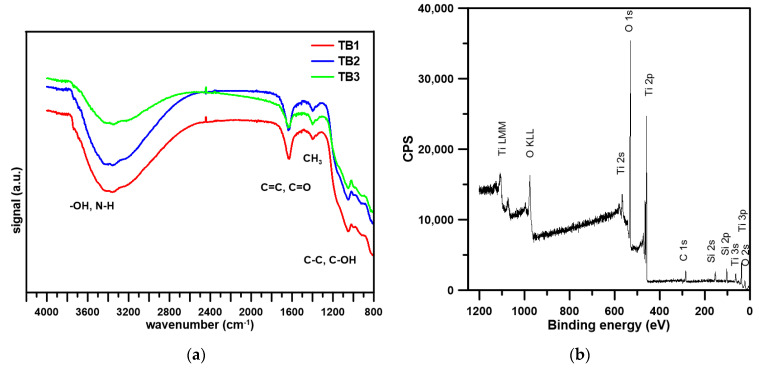
Physicochemical characteristics of tested: (**a**) FTIR spectra; (**b**) XPS spectrum of TB2.

**Figure 3:**
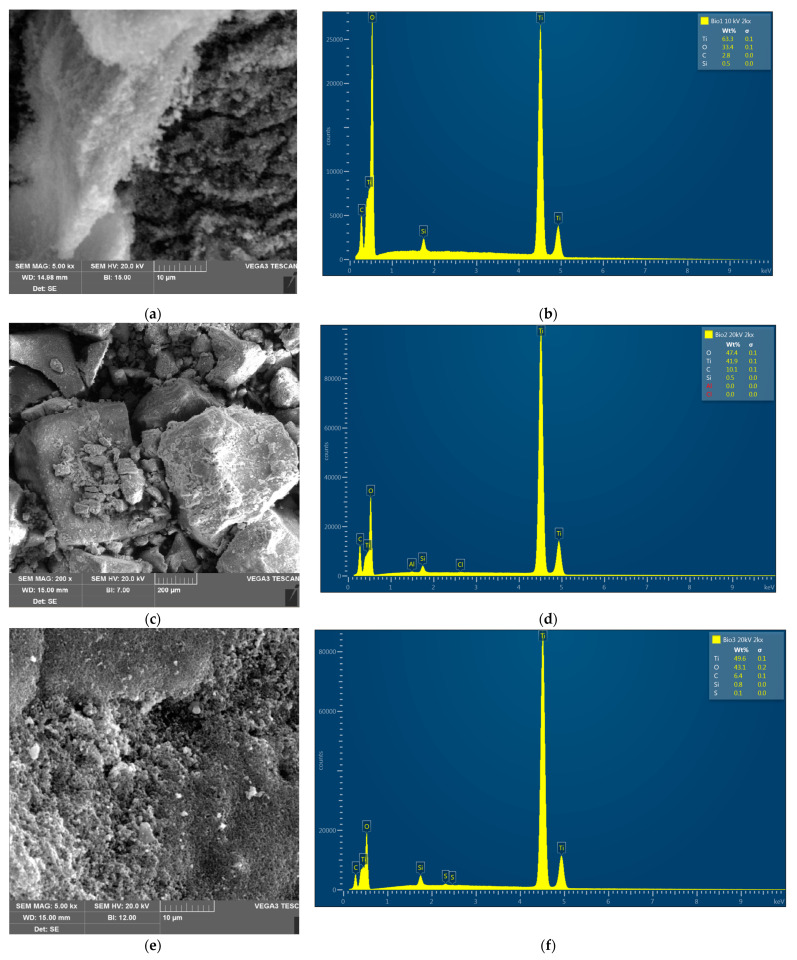
SEM images of the photocatalysts: (**a**) TB1 with magnification 5 kx; (**b**) EDS spectra of TB1; (**c**) TB2 with magnification 200×; (**d**) EDS spectra of TB2; (**e**) TB3 with magnification 5 kx; (**f**) EDS spectra of TB3.

**Figure 4:**
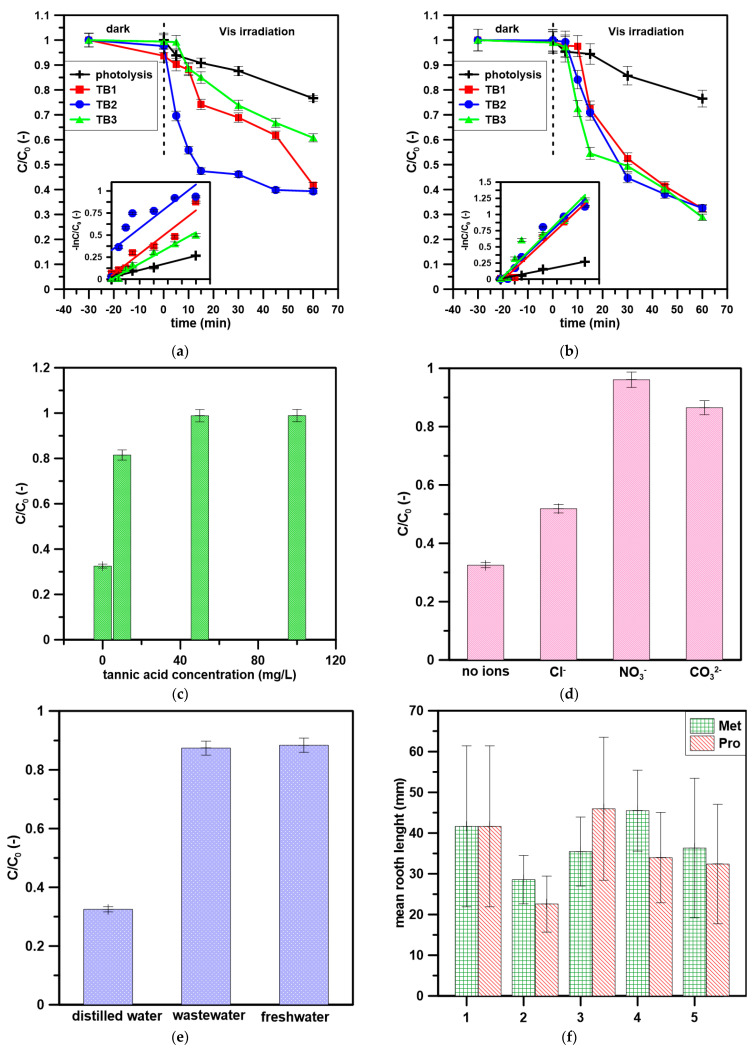

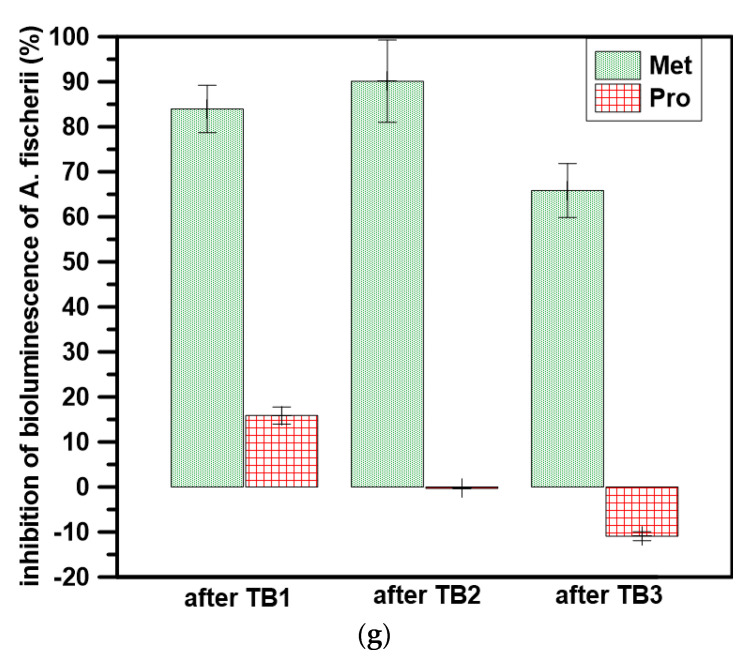
The removal of Met and Pro: (**a**) Met removal kinetics, inside: pseudo-first-order fitting; (**b**) Pro removal kinetics, inside: pseudo-first-order fitting; (**c**) DOM effect; (**d**) inorganic ions effect [c_0_ = 0.001 M]; (**e**) effect of water matrix; (**f**) PHYTOTOXKIT test results; (**g**) Microtox^®^ test results.

The obtained photocatalysts were subjected to physicochemical characterization to analyze their surface morphology, composition, and photocatalytic activity. The results were presented in Table 1 and Table 2 and Figure 2, Figure 3 and Figure 4.

### 3.1. Physicochemical Properties of the Photocatalysts

#### Surface Properties

The physicochemical analysis of the biochar was performed previously [41]. Briefly, the presence of O–H, N–H, carbonyl structures, and aryl and vinyl functionalities was evidenced by XPS and FT-IR spectra, implying the increased active centers for adsorption and surface reactions. All the obtained photocatalysts were characterized by similar surface area, about 192 m^2^/g (Table 1), and similar pore diameter (2.54–2.65 nm). However, the photocatalysts differ in surface composition. The surface of TB was containing C%, O%, Ti%, and traces of Si as the result of TEOS application during preparation). Firstly, the content of C% on the surface of the photocatalyst was very broad, and in the case of TB1, only 2.8% of C% was on the surface which may indicate that the hardwood-derived biochar behaved as a support of the photocatalyst. TB2 was characterized by the highest O% on the surface that can participate in the formation of bonds with Met or Pro [42]. The highest content of carbon was noted in TB2 (Table 1). The photocatalysts were characterized by the presence of -OH groups on the surface (peak at ~ 4000–3200 cm^−1^) The presence of biochar (C=C, C=O functionalities) was evidenced in FTIR spectra (Figure 2a) [43]. XPS studies (Figure 2b) confirmed the presence of Ti (as TiO_2_) [22].

The morphology of the photocatalysts was determined in scanning electron microscopy and the images with various magnifications were presented in Figure 3. It can be seen that the structure of TB1 is crystalline and compact (Figure 3a), while higher magnification shows that the surface is heterogeneous and wavy, resembling cotton wool (Figure 3b). This suggests a porous structure. The morphology of TB2 (when biochar from sunflower was used) is similar, but the photocatalyst is more crumbly, and higher magnification shows that the surface is flatter, but also rough, which may favor adsorption capacity. The surface of TB3 is also crystalline and rough, although the surface is less regular than with previous materials. The porous structure of the C–TiO_2_ composite catalyst can improve the adsorption ability towards organic compounds [22].

### 3.2. Photocatalytic Oxidation of Met and Pro

#### 3.2.1. Kinetics

Photolysis, i.e., removal under the action of light alone, allowed only about 20% reduction in the concentration of metoprolol, which indicates that this compound will not be effectively degraded under the influence of sunlight (Figure 4a,b). After 30 min of darkness, the greatest decrease in metoprolol concentration was observed using TB1 (Figure 4a), amounting to just over 6%. This indicates a rather weak sorption capacity of the tested photocatalyst. The other photocatalysts showed an even lower ability to absorb metoprolol, the smallest loss of concentration was observed for the TB3, which adsorbed less than 1% of the drug.

After switching on the lamp, samples were taken at specific time intervals, which allowed for a detailed determination of the decrease in metoprolol concentration from the solution during irradiation. The photocatalytic reaction proceeded the fastest using TB2 (Figure 4a). Already after 15 min of the irradiation, only 47% of Met remained. The subsequent process allowed the removal of a relatively small amount of the drug. This proves that products resistant to further photodecomposition were formed during photo-oxidation. Each photocatalyst improved the efficiency of the process of Met removal, but only TB1 and TB2 removed approximately 60% of Met, although they needed different times for this. TB2 turned out to be the most effective due to the speed and percentage of the drug removed. However, it cannot be excluded that TB1 would remove more Met if the process was run longer because the shape of the concentration change curves. About 80% removal of Met was noted in [14], where BioMnOx/PMS system using BioMnOx-coated biofilm carriers as a specific catalyst was used. Our results were lower than observed in [18]; however, the authors used LaCoO_3_/graphene catalyst but with an additional peroxymonosulfate activation system (Table 2). Although some authors observed similar or higher removal rates (Table 2), their methods required energy-consuming UV light or additional procedures (like PMS as a second oxidizing agent). The results clearly indicate that the application of novel biochar-TiO_2_ photocatalysts irradiated by visible light is a promising method of β-blockers removal from water.

For the propranolol solution (Figure 4b), practically no adsorption process was observed on the tested materials. After switching on the lamp, the largest loss of propranolol concentration in the shortest time was recorded for TB3, 54% of Pro remained after 15 min after the irradiation, and the further process allows for the removal of an additional approx. 30% of the tested drug. However, all 3 photocatalysts show similar efficiency: loss of propranolol concentration is about 70%.

The kinetics of Met and Pro removal followed the pseudo-first-order regime [44]. Table 3 presents kinetic data: k_1_—pseudo-first-order rate constant, T_1/2_—the half-life of the compound, and R^2^—fitting to the pseudo-first-order kinetic model. The fastest removal during the first 15 min of treatment of Met was presented by the process involving TB2, and the slowest by the photolysis process. Observing the value of the rate constant, it can be clearly stated that the most effective photocatalyst was the photocatalyst with the addition of sunflower biochar. The results imply that biochar was mainly the support for the incorporation of TiO_2_. However, the increased surface area and sensitizing effect of biochar in biochar-TiO_2_ photocatalysts cannot be excluded. Biochar in the photocatalysts is acting as an electron reservoir and increases charge separation which results in increased photocatalytic activity. The reduced band gap energy of biochar-TiO_2_ photocatalysts indicates visible light activation [45].

#### 3.2.2. Effect of Matrix Parameters

Tannic acid was used to illustrate how organic dissolved matter can affect the photocatalytic process (Figure 4c). It is clearly visible that with the increase in the concentration of tannic acid, the photocatalytic ability of the photocatalyst decreases, and the process is less and less efficient. Already for a concentration of 10 mg/L (which is usually present in natural waters), the efficiency of the process is low. It can be concluded that tannic acid blocks the access of light to the photocatalyst or may compete with Met or Pro molecules and photocatalysts’ surface [46]. Additionally, it was observed that tannic acid may act as a radical scavenger and in this way lower the efficiency of the photocatalytic reaction [47]. Also, the presence of inorganic ions such as Cl^−^, NO_3_^−^, and CO_3_^2−^ ions in water affects the photocatalytic process (Figure 4d). The process is most strongly inhibited by NO_3_^−^ ions, followed by CO_3_^2−^.

The water used for photocatalysis also affects the efficiency of photocatalytic oxidation using tested materials (Figure 4e). The best results were achieved for the distilled water sample because there were no other impurities in it, so only the pharmaceutical was degraded. The treated wastewater used for the process, which is discharged into the environment, does not bring satisfactory results during the photocatalysis process. There are a lot of other impurities in it that block the efficiency of the photocatalytic process. A similar effect is exerted by river water from the Dnieper River (Ukraine). The obtained results have shown that although the removal in distilled water was efficient, the application of a slightly complicated matrix would lower the process efficiency [48]. The reusability test was performed and washing with methanol and distilled water was performed and revealed that the activity was reduced by 20% after the 3rd run.

#### 3.2.3. Toxicity

In (Figure 4f,g), the results of toxicity to the tested plant and bacteria are presented. The length of the roots of the plant was measured because seed germination begins with root growth so that the plant can take up water and nutrients. The control trial was designed to show how watercress grows in a standard environment. The test showed that for water before the photocatalytic process, the average root length was 29 mm for metoprolol and 23 mm for propranolol. This is 14 mm less for the metoprolol solution and 19 mm less for the propranolol solution. Subsequent tests show that the photocatalysis process has a positive effect on plant development. The most effective catalysts are TB1 and TB2, for them the roots of the plant are much longer, even than for the control sample. The Microtox^®^ test results indicated that bacteria were more sensitive to the presence of samples after photocatalytic treatment of Met (Figure 4g) and the inhibition of the bacteria bioluminescence was more than 80% hindered independently from the used photocatalyst. In the case of Pro, the results were slightly opposite: no toxicity was noted to bacteria after photocatalytic treatment. The results indicate that using only a decrease in the tested pollutant concentration is not effective enough in establishing the treatment method’s safety. There is a need to use additional testing such as ecotoxicity tests; however, the key parameter is the properly chosen tested organism.

#### 3.2.4. PALS Studies

**Figure 5:**
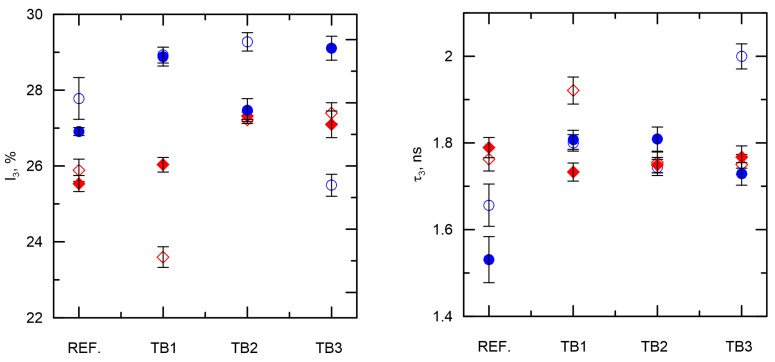
The o-Ps intensity (I_3_) and lifetime (τ_3_) for reference and samples with different photocatalysts during removal of Met (dots) and Pro (diamonds) in darkness (empty points) and light (full points).

The process of water purification from the Met and Pro in the dark and light in the absence of photocatalysts is reflected by the lifetime and intensity parameters of o-Ps. As shown in Figure 5, after turning on the light, a decrease in the I_3_ intensity is observed for both Met and Pro. At the same time, the lifetime τ_3_Met_ (blue dots) is shorter than in the τ_3_Pro_ (red diamonds), due to an additional aromatic ring in Pro. The lifetime τ_3_ in pure water (not degassed, containing paramagnetic oxygen molecules, O_2_) is 1.8 ns [49]. Removal of paramagnetic molecules from the liquid leads to the extension of the o-Ps lifetime up to 1.902 ns [33,50,51]. From the point of view of the changes recorded by the o-Ps probe, the efficiency of the Met photolysis process in the presence of TB1 in the dark and on the illuminated sample is similar (in time scale = 1 h); however, in the case of Pro removal, lighting leads to a significant increase in I_3_ intensity. The higher I_3_ intensity can be explained by the higher probability of o-Ps formation, which is favored by the elimination of radical products from the liquid medium. In the presence of TB2, the efficiency of Met and Pro purification in the presence of light is similar, and in addition, the final τ_3_ result obtained is close to that known for pure water. The use of TB3 leads to a reduction of τ_3_ below the value measured in pure water, regardless of the presence of pollutants (Met or Pro).

The lifetime of o-Ps in the degraded metoprolol solution for TB2 increased with the decreasing concentration of the pharmaceutical. This proves the change in the concentration of ions in the sample under the influence of light. For the Pro solution, the key role in the obtained PALS result is played by the speed of the purification process in the presence of catalysts. For TB1, the time needed to place the sample in the measuring chamber is shorter than the time needed to initiate the photocatalysis process; therefore, the PALS technique can distinguish between drug concentrations in a sample stored in the dark and in an illuminated one. For TB2 and TB3, the photocatalysis process begins immediately after the light is turned on, so in the samples placed in the PALS chamber, the photocatalysis process was activated at the time of pouring the samples into the chamber. This is a factor probably responsible for the high agreement of the results of all PALS parameters for samples measured in the light and the dark.

## 4. Conclusions

The research results indicate that the most effective photocatalyst for removing β-blockers may be TB2 with an admixture of sunflower biochar. It needs visible light in the 400–800 nm range to activate. The sol-gel method of obtaining the photocatalyst had an impact on the surface of the materials. Based on the elemental composition, it can be concluded that the photocatalyst with a greater admixture of carbon and oxygen is more effective. The regular porous structure has a positive effect on adsorption and photocatalytic abilities. Carbon added to TiO_2_ acted as the support for TiO_2_ and enhanced the activity in visible light. Studies have shown that its amount and the structure itself, which must be regular and porous, play an important role. TB1 also shows good efficiency, the half-life of the pollutant is equal for both materials. However, the rate constant is the highest for TB2.

Studies have shown that other impurities, such as organic compounds or ions of inorganic compounds, have a large impact on the water purification process because they block the effectiveness of the process. Photocatalytic compounds have a positive effect on the development of plants and the toxicity of water entering the soil. Tap water itself contains impurities that inhibit plant growth, and TB1 and TB2 can be used in the purification of water used for plant cultivation.

PALS results largely coincide with the results of photocatalytic processes. The factor determining the lifetime of the orthopositronium was the surface tension of the drug solution. The highest agreement between the PALS parameters of pure water and water treated with Met and Pro was obtained with the use of TB2.

As shown by the data and the high value of k_1_-, the reaction rate is the highest for TB2. For this reason, the material used on a larger scale can be TiO_2_ with an admixture of sunflower biochar. However, it is necessary to consider its weaker effect on sewage and river waters. Therefore, there is a probability that before carrying out such a process of water purification from pharmaceuticals, the water will first have to pass through a classic wastewater treatment plant, so that the concentration of organic compounds and inorganic ions does not inhibit the process. The photocatalyst itself does not wear out and can be reused, and the use of daylight to activate the process reduces the cost of cleaning up pollutants.

## Figures and Tables

**Table 1:** The physicochemical properties of tested materials.

Photocatalysts	S_BET_ [m^2^/g]	V_p_ [cm^3^/g]	D [nm]	C ^1^ [%]	O ^1^ [%]	Ti ^1^ [%]
TB1	192	0.0268	2.60	2.8	33.4	63.3
TB2	192	0.0483	2.54	10.1	47.4	41.9
TB3	192	0.0172	2.65	6.4	43.1	49.6

S_BET_—surface area, V_p_—pore volume, D—pore diameter, ^1^ from EDS mapping.

**Table 2:** Comparison of the obtained results with the literature data.

Β-Blocker	Material	Efficiency	Reference
Met	Biochar-TiO_2_	60%, Vis	Our studies
	BioMnOx	80%, PMS	[14]
	LaCoO_3_/graphene	100%, PMS	[18]
	TiO_2_	60%, UV	[36]
	B-TiO_2_	90%, UV	[37]
Pro	Biochar-TiO_2_	70%, Vis	Our studies
	TiO_2_	70%, solar	[38]
	Nd–TiO_2_	95%, UV	[39]
	carbon dot/TiO_2_	99%, UV-Vis	[40]

PMS—peroxymonosulfate.

**Table 3:** Kinetics of Met and Pro removal.

Photocatalysts	k_1_ [min^−1^] ×10^−3^	Met T_1/2_ [min]	R^2^ [-]	k_1_ [min^−1^] ×10^−3^	Pro T_1/2_ [min]	R^2^ [-]
Photolysis	4.05	171	0.9686	4.38	158	0.9813
TB1	12.33	56	0.9372	20.27	34	0.9726
TB2	12.42	56	0.7073	20.54	34	0.9539
TB3	8.51	81	0.9762	19.75	35	0.9302

## Data Availability

The data presented in this study are available on request from the corresponding author.
